# Spontaneous Unruptured Middle Cerebral Artery Dissection Presenting With Ischemia: Conservative Management and Rescue Endovascular Intervention

**DOI:** 10.7759/cureus.105745

**Published:** 2026-03-24

**Authors:** Ryo Matsuzaki, Yutaka Fuchinoue, Sho Nitta, Masaaki Nemoto, Nobuo Sugo

**Affiliations:** 1 Department of Neurosurgery, Faculty of Medicine, Toho University, Tokyo, JPN; 2 Department of Neurosurgery, Faculty of Medicine, Toho University, Chiba, JPN

**Keywords:** conservative vs surgical management, dual antiplatelet therapy (dapt), endovascular reconstruction, spontaneous isolated middle cerebral artery dissection, wingspan stent

## Abstract

Spontaneous middle cerebral artery (MCA) dissection is a rare cause of ischemic stroke. Due to the lack of established guidelines, management strategies, ranging from conservative medical therapy to endovascular reconstruction, remain controversial. In Case 1, a 53-year-old woman presented with acute left upper limb paresis. Imaging revealed an infarct in the right MCA territory and angiographic features of dissection. She was treated conservatively with dual antiplatelet therapy. Her symptoms resolved, and follow-up imaging at 10 weeks demonstrated spontaneous angiographic healing of the vessel. In contrast, Case 2 involved a 72-year-old man who presented with right upper limb weakness due to left MCA dissection. Initial conservative management with antiplatelet therapy was insufficient; the patient experienced neurological deterioration with expanding infarction on day 5. Emergent endovascular reconstruction using a Wingspan stent (Stryker Neurovascular, Fremont, CA, USA) was performed. The procedure successfully stabilized the dissection, improved distal perfusion, and halted further neurological decline. These two cases suggest that conservative management may be reasonable in selected clinically stable patients with spontaneous unruptured ischemic MCA dissection, whereas rescue endovascular stenting may be considered in patients with neurological worsening and imaging progression despite medical therapy. Because this report includes only two cases, these observations should be interpreted cautiously.

## Introduction

Spontaneous intracranial artery dissection is an increasingly recognized cause of stroke in young and middle-aged adults. While dissections of the vertebrobasilar system are relatively common, isolated dissection of the middle cerebral artery (MCA) is rare [1]. In a systematic review of 61 cases of isolated MCA dissection, ischemia was the most common presentation, followed by hemorrhage [1]. The clinical presentation varies widely, from headache and ischemic symptoms to subarachnoid hemorrhage (SAH), depending on the plane of dissection (subintimal versus subadventitial) [2].

Because of its rarity, optimal treatment strategies for spontaneous unruptured ischemic MCA dissection are not well standardized. Conservative management with antithrombotics is generally favored for stable patients to promote spontaneous healing [3]. However, in cases of hemodynamic compromise or progressive thrombosis, medical management may fail. In such refractory cases, endovascular intervention, including angioplasty or stenting, has been proposed as a rescue therapy, though evidence is limited to case reports and small series [4].

We herein present two cases of spontaneous unruptured MCA dissection presenting with ischemia, with contrasting clinical courses: one achieving spontaneous angiographic resolution with conservative care and another requiring urgent intracranial stenting (Wingspan, Stryker Neurovascular, Fremont, CA, USA) due to progressive neurological deterioration. By presenting these contrasting trajectories, we aim to illustrate how serial clinical and imaging findings may inform individualized management, rather than to propose a treatment algorithm.

## Case presentation

Case 1: spontaneous resolution with conservative management

A 53-year-old woman with a history of Hashimoto’s disease presented to our emergency department with sudden difficulty moving her left hand and dysarthria. She had no history of head trauma or neck manipulation. On admission, her Glasgow Coma Scale (GCS) score was 15. Neurological examination revealed left central facial palsy and left upper limb paresis (Manual Muscle Test grade 3/5). The National Institutes of Health Stroke Scale (NIHSS) score was 2 [5].

**Figure 1:**
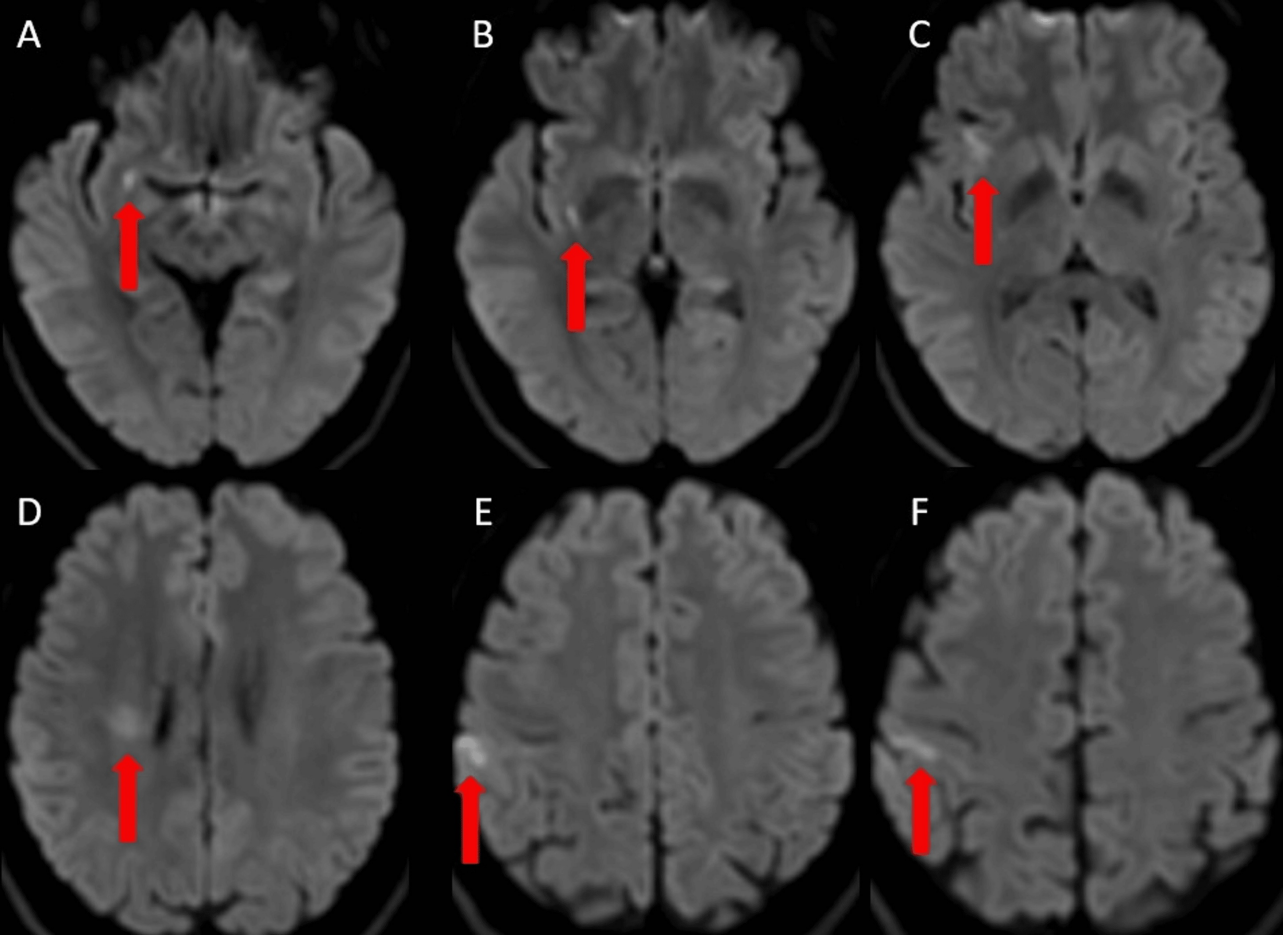
Case 1: Initial MRI on admission (A-F) DWI demonstrating multiple acute ischemic infarcts scattered throughout the right MCA territory (red arrows). DWI, diffusion-weighted imaging; MCA, middle cerebral artery

**Figure 2:**
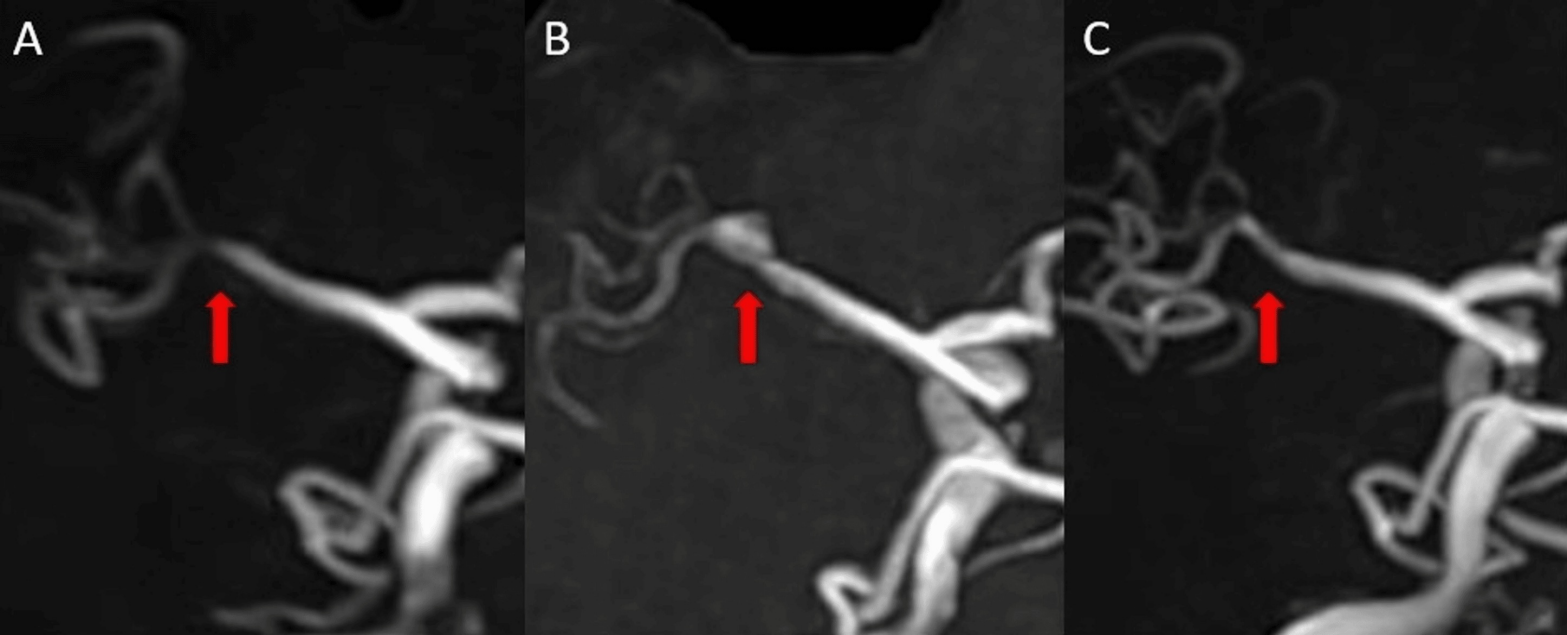
Case 1: Serial MRA showing the clinical course (A) MRA on admission showing stenosis and irregularity of the right MCA (arrow). (B) MRA at three weeks after onset showing the “pearl and string” sign (arrow). (C) MRA at 10 weeks after onset demonstrating spontaneous resolution and normalization of the vessel caliber (arrow). MCA, middle cerebral artery; MRA, magnetic resonance angiography

MRI demonstrated acute scattered infarcts in the right MCA territory (Figure 1A-1F). Magnetic resonance angiography (MRA) on initial presentation demonstrated stenosis of the right MCA M1 segment (Figure 2A). The patient was admitted and treated conservatively with dual antiplatelet therapy (aspirin 100 mg/day and clopidogrel 75 mg/day) and a free radical scavenger (edaravone). Dual antiplatelet therapy was selected because the patient was relatively young and considered to have a low hemorrhagic risk based on her clinical background.

**Figure 3:**
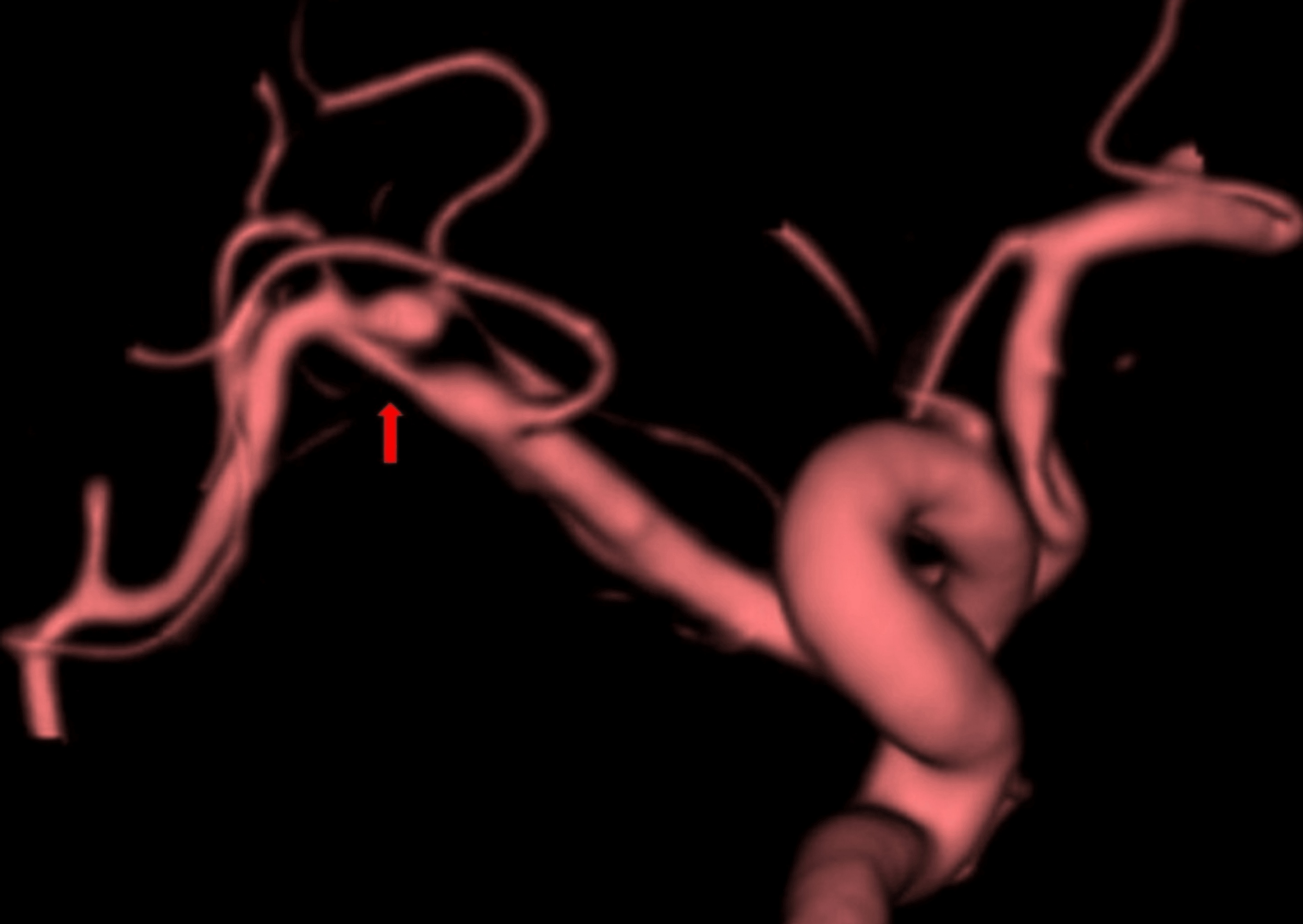
Case 1: 3D-RA performed three weeks after onset The image reveals the characteristic “pearl and string” sign (arrow), strongly suggestive of arterial dissection. 3D-RA, three-dimensional rotational angiography

Her symptoms gradually improved without fluctuation. A follow-up MRA performed three weeks after onset revealed a persistent “pearl and string” sign (Figure 2B), which was further confirmed by three-dimensional rotational angiography (Figure 3). She was discharged home with a modified Rankin Scale (mRS) score of 0 [6,7]. Serial MRA follow-up was performed. Imaging at five and 10 weeks showed a gradual improvement of the stenosis. The final follow-up MRA at 10 weeks demonstrated complete normalization of the vessel caliber, confirming spontaneous healing of the dissection (Figure 2C). The patient remained symptom-free during the follow-up period. 

Case 2: progressive ischemia requiring Wingspan stenting

**Figure 4:**
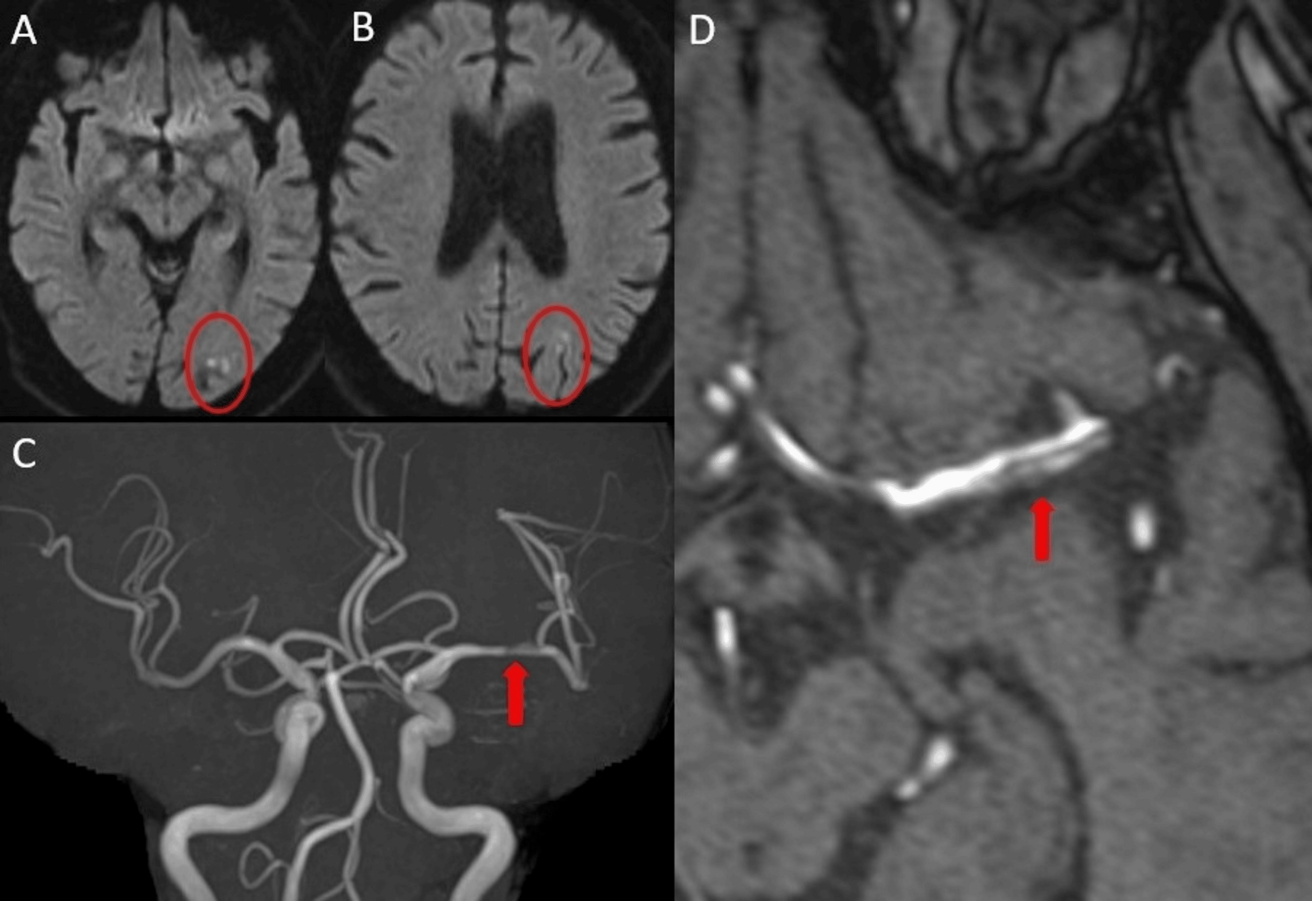
Case 2: Initial imaging on admission (A, B) DWI showing acute ischemic stroke in the left cerebral watershed area (red ovals). (C, D) MRA demonstrating findings suggestive of dissection in the horizontal (M1) segment of the left MCA (arrow). DWI, diffusion-weighted imaging; MCA, middle cerebral artery; MRA, magnetic resonance angiography

A 72-year-old man with a history of hypertension, diabetes mellitus, and prior cerebral infarction presented with subjective weakness in his right upper limb upon awakening. On admission, he was alert with mild right hemiparesis. MRI revealed acute ischemic changes in the left MCA territory (Figure 4A, 4B). MRA demonstrated severe stenosis of the left MCA trunk (Figure 4C, 4D). Intravenous rt-PA was not administered because more than 4.5 hours had elapsed since symptom onset.

**Figure 5:**
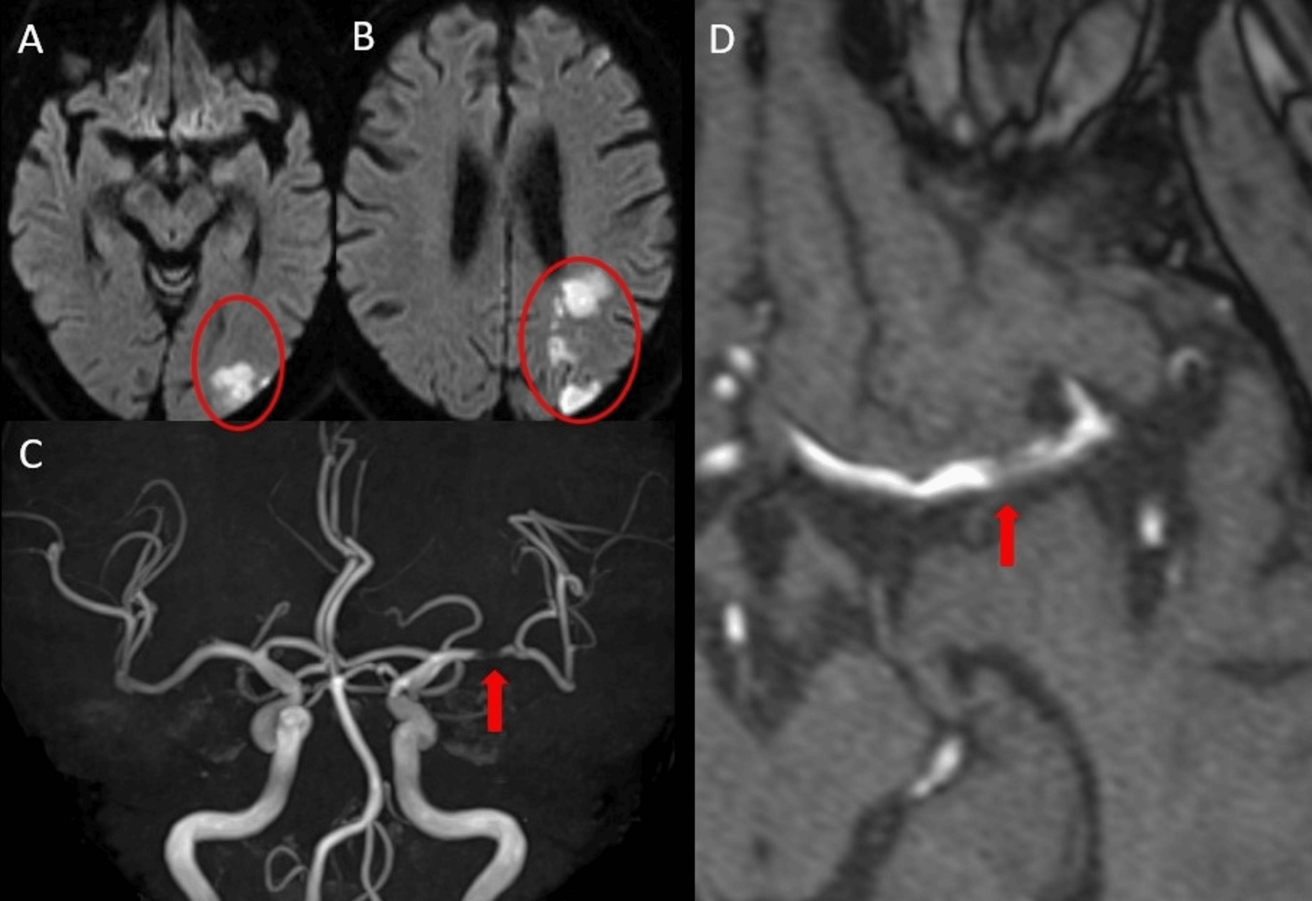
Case 2: Imaging progression on hospital day 5 (A, B) Follow-up DWI showing exacerbation and expansion of the known infarcts following clinical deterioration (red ovals). (C, D) MRA demonstrating progression of the dissection and stenosis compared to admission imaging (arrow). DWI, diffusion-weighted imaging; MRA, magnetic resonance angiography

**Figure 6:**
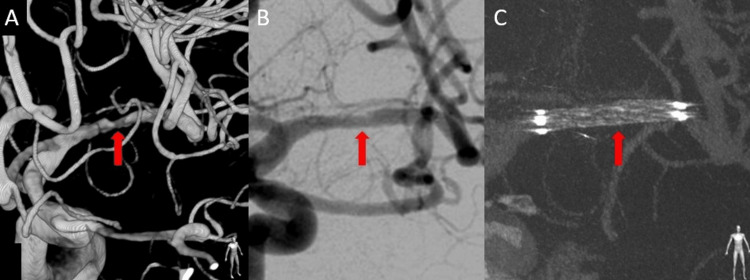
Case 2: Endovascular reconstruction using the Wingspan stent system (A) Pretreatment 3D-RA showing the dissected segment (arrow). (B) Posttreatment 2D DSA showing restoration of true lumen patency (arrow). (C) Posttreatment 3D-RA confirming good wall apposition of the stent (arrow). 3D-RA, three-dimensional rotational angiography; DSA, digital subtraction angiography

He was initially managed conservatively with antiplatelet therapy (clopidogrel 75 mg/day) and intravenous Ozagrel sodium (a thromboxane A₂ synthase inhibitor). However, on hospital day 5, he developed sudden worsening of right hemiparesis and motor aphasia. Repeat MRI showed expansion of the ischemic core in the left MCA territory (Figure 5A, 5B) and progression of the stenosis (Figure 5C, 5D). Digital subtraction angiography (DSA) was performed urgently, confirming a flow-limiting dissection of the left MCA M1 segment with delayed distal perfusion (Figure 6A). 

Given the failure of medical management and clinical progression, endovascular reconstruction was indicated. Under local anesthesia, a Wingspan stent, a self-expanding nitinol stent designed for intracranial atherosclerotic disease, was navigated to the lesion and deployed across the dissected segment. Post-procedural angiography demonstrated excellent wall apposition of the stent, restoration of true lumen patency (Figure 6B), and significant improvement in distal blood flow (Figure 6C). Following the procedure, his neurological deterioration was halted. He was maintained on dual antiplatelet therapy to prevent in-stent thrombosis.

MRI performed within one week after treatment showed no new abnormalities and no evidence of perforator infarction. Delayed vascular imaging follow-up was performed with postoperative MRA only; CT angiography (CTA) and DSA were not performed. At discharge, the patient’s NIHSS score was 6, and the mRS score was 3. Although he had residual deficits, he was discharged to a rehabilitation facility in stable condition.

## Discussion

These two cases highlight the spectrum of clinical behaviors in spontaneous unruptured MCA dissection presenting with ischemia and underscore the necessity of a flexible treatment approach. Spontaneous MCA dissection is distinct from extracranial dissection: intracranial arteries lack an external elastic lamina and have a thinner adventitia, making them more susceptible to rupture or dissection [8].

Conservative management and spontaneous healing

For patients presenting with ischemia who are hemodynamically stable, conservative management is the standard of care. Case 1 demonstrates the natural history of a “benign” dissection, in which the intramural hematoma is gradually reabsorbed, leading to restoration of the vessel lumen. Several reports have documented this phenomenon of “spontaneous angiographic resolution” within three to six months [9,10]. In such cases, invasive procedures, which carry inherent risks of perforation or occlusion of perforating arteries, should be avoided. Serial noninvasive imaging (MRA or CTA) is crucial to monitor for vessel healing or aneurysm formation.

Role of endovascular intervention

Conversely, Case 2 illustrates a “malignant” course characterized by hemodynamic instability. The deterioration on day 5 was likely due to expansion of the intramural hematoma or flap closure, compromising distal flow. In this scenario, medical therapy alone is insufficient. Endovascular options include balloon angioplasty, stent placement, or mechanical thrombectomy. While balloon angioplasty alone carries a risk of recoil or vessel rupture, stent placement provides radial force to tack down the intimal flap and maintain luminal patency [11].

We utilized the Wingspan stent system in Case 2. Although the Wingspan stent is FDA-approved for intracranial atherosclerotic stenosis refractory to medical therapy, its use in dissection is off-label. However, its high flexibility and lower radial force compared with balloon-mounted stents make it suitable for navigating tortuous intracranial vessels and treating dissection with a lower risk of iatrogenic rupture [12].

In Case 2, atherosclerotic MCA stenosis, embolic occlusion, and other vasculopathies were considered in the differential diagnosis. The diagnosis of dissection was supported by evolving luminal morphology on serial imaging and by urgent DSA demonstrating a flow-limiting dissected segment. Our experience suggests that Wingspan stenting may be a viable rescue strategy for MCA dissection when medical management fails.

Antithrombotic considerations

The optimal antithrombotic strategy for ischemic MCA dissection remains uncertain. In Case 1, dual antiplatelet therapy was selected because the patient was relatively young and considered to have a low hemorrhagic risk. In Case 2, single antiplatelet therapy was initially used during conservative management, and dual antiplatelet therapy was instituted after stent placement to reduce the risk of in-stent thrombosis. Our two cases do not allow conclusions regarding the superiority of single antiplatelet therapy, dual antiplatelet therapy, or anticoagulation.

In clinically stable unruptured ischemic cases without neurological or imaging progression, invasive treatment should be avoided because spontaneous healing may occur, and procedural risks include vessel injury or occlusion of perforating arteries. When endovascular reconstruction is not feasible or appropriate, surgical options may need to be considered; however, our two cases do not permit firm conclusions regarding surgical indications.

Limitations

This report is limited by its retrospective nature and small sample size. The optimal duration of antiplatelet therapy and the long-term patency of stents in dissection cases require further investigation. In addition, this report has limited generalizability and is subject to the publication bias inherent to illustrative case reports. High-resolution vessel wall MRI, which can directly visualize the intramural hematoma, was not available for these cases at the time of presentation [13]; therefore, the diagnosis relied on serial luminal imaging, angiographic findings, and clinical course.

## Conclusions

Spontaneous MCA dissection requires a dynamic treatment strategy based on the clinical course. Conservative management with antiplatelet therapy is effective for stable patients, often resulting in spontaneous angiographic resolution. However, for patients with progressive symptoms or hemodynamic compromise, urgent endovascular reconstruction using a self-expanding stent (e.g., Wingspan) may be considered as a rescue treatment option. Early recognition of clinical fluctuation is key to timely therapeutic escalation.
